# Stepwise mechanism for transcription fidelity

**DOI:** 10.1186/1741-7007-8-54

**Published:** 2010-05-07

**Authors:** Yulia Yuzenkova, Aleksandra Bochkareva, Vasisht R Tadigotla, Mohammad Roghanian, Savva Zorov, Konstantin Severinov, Nikolay Zenkin

**Affiliations:** 1 Centre for Bacterial Cell Biology, Institute for Cell and Molecular Biosciences, Newcastle University, Newcastle upon Tyne, NE2 4AX, UK; 2 Department of Physics, Boston University, Boston, MA 02215, USA; 3 Faculty of Bioengineering and Bioinformatics, Moscow State University, Moscow, Russia; 4 Waksman Institute, Rutgers, the State University of New Jersey, Piscataway, NJ 08854, USA; 5 Department of Molecular Biology and Biochemistry, Rutgers, the State University of New Jersey, Piscataway, NJ 08854, USA; 6 Institute of Molecular Genetics, Russian Academy of Sciences, Moscow, Russia

## Abstract

**Background:**

Transcription is the first step of gene expression and is characterized by a high fidelity of RNA synthesis. During transcription, the RNA polymerase active centre discriminates against not just non-complementary ribo NTP substrates but also against complementary 2'- and 3'-deoxy NTPs. A flexible domain of the RNA polymerase active centre, the Trigger Loop, was shown to play an important role in this process, but the mechanisms of this participation remained elusive.

**Results:**

Here we show that transcription fidelity is achieved through a multi-step process. The initial binding in the active centre is the major discrimination step for some non-complementary substrates, although for the rest of misincorporation events discrimination at this step is very poor. During the second step, non-complementary and 2'-deoxy NTPs are discriminated against based on differences in reaction transition state stabilization and partly in general base catalysis, for correct versus non-correct substrates. This step is determined by two residues of the Trigger Loop that participate in catalysis. In the following step, non-complementary and 2'-deoxy NTPs are actively removed from the active centre through a rearrangement of the Trigger Loop. The only step of discrimination against 3'-deoxy substrates, distinct from the ones above, is based on failure to orient the Trigger Loop catalytic residues in the absence of 3'OH.

**Conclusions:**

We demonstrate that fidelity of transcription by multi-subunit RNA polymerases is achieved through a stepwise process. We show that individual steps contribute differently to discrimination against various erroneous substrates. We define the mechanisms and contributions of each of these steps to the overall fidelity of transcription.

## Background

**Figure 1:**
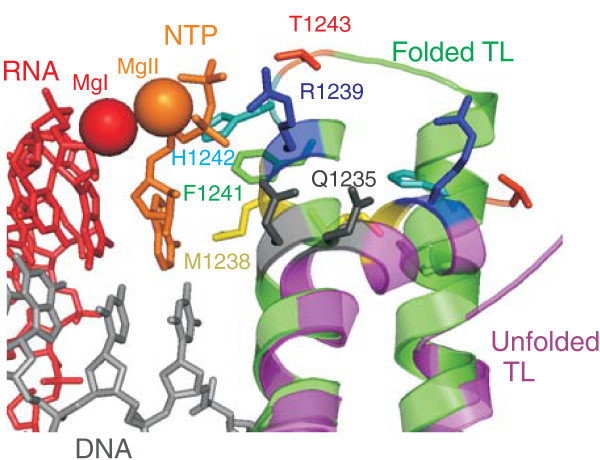
**Folding of the Trigger loop (TL) restructures an amino acid content of the active centre**. The active centre of the *Thermus thermophilus *RNAP elongation complex with bound substrate is shown (there is complete conservation of sequence in TL and active site residues between *T. thermophilus *and *T. aquaticus *(used in this study) RNAPs). Unfolded TL (magenta) and folded TL (green; PDB 2PPB and 2O5J, respectively [9]) in the active centre are shown as ribbons. The amino acids of the TL that face the nucleotide triphosphate (NTP; orange) bound in the i+1 site upon TL folding, and that were analysed in our study, are shown as coloured sticks (Q1235 grey, M1238 yellow, R1239 blue, F1241 green, H1242 cyan, T1243 red) in both folded and unfolded states of TL. The NTP in i+1 site is orange, RNA is red and template DNA in grey. Mg^2+ ^ions of the active centre are shown as spheres.

All reactions performed by RNA polymerase (RNAP; phosphodiester bond synthesis, pyrophosphorolysis, phosphodiester bond hydrolysis) are catalysed by a single active centre and are proposed to proceed through a general two Mg^2+ ^ion mechanism [1-4]. Structural studies revealed that conformational changes in the active centre might be required for efficient catalysis and biochemical studies are consistent with such a model [5-11]. The major conformational change involves folding of the Trigger Loop (TL) that brings the RNAP active centre from 'open' to 'closed' conformation (Figure 1). Though crystal structures of RNAPs with folded TL were obtained only in the presence of cognate NTP (cNTP) bound in the i+1 site of the active centre, TL folding is also thought to be required for efficient catalysis of pyrophosphorolysis and phosphodiester bond hydrolysis [7]. Although, in the absence of TL, the rate of phosphodiester bond formation is decreased by four orders of magnitude, the actual mechanism by which TL participates in RNAP activity remains unknown. Crystallographic studies revealed that folded TL tightly interacts with cNTP base and triphosphate moieties [9,11] (Figure 1). This observation was used to build three distinct models explaining the role of TL folding in phosphodiester bond formation. In the first model, based on a crystal structure of yeast RNAP II, folded TL was proposed to directly participate in catalysis through an invariant histidine residue (β' H1242, here and throughout the text *Thermus aquaticus *RNAP numbering is used) that withdraws electron density from the β-phosphate of the incoming NTP thus activating nucleophilic attack on the α-phosphorus [11]. According to the second model H1242 participates in acid catalysis as a proton donor for the leaving pyrophosphate [12]. This model is based on studies of single-subunit RNAPs that are evolutionary unrelated to multi-subunit RNAPs and may, therefore, use a different mechanism for catalysis. In the third model, based on a crystal structure of *T. thermophilus *RNAP, folding of TL was proposed in order to stabilize, via R1239 and H1242 residues, the triphosphate moiety of the i+1 site-bound NTP in the active (insertion) conformation (transition state stabilisation or orientation catalysis) [7,9]. A recent study of *Escherichia coli *RNAP revealed that mutation of H1242 had only minor effect on catalysis, indicating that acid/base catalysis is not its primary role [13]. However, the relatively mild effects of substitutions of amino acids that, according to crystallographic data, interact with the triphosphate moiety of incoming NTP observed in this work (R1239A, H1242A, R1239A/H1242A) are also inconsistent with the transition state stabilisation hypothesis [9]. The small effects of single substitutions, compared to dramatic catalytic defect caused by deletion of the entire TL, suggest that TL may have an allosteric effect on other amino acids that are also required for catalysis (see Discussion).

Mutations in TL and surrounding amino acids were found to alter discrimination against non-complementary NTP (ncNTP) and complementary 2'- and 3'-deoxy NTPs (c2'dNTPs and c3'dNTPs) [6,10,11,13,14]. These observations led to suggestions that TL could participate in substrate discrimination by either: (i) selectively facilitating incorporation of correct substrates; or (ii) by providing sufficient time for accurate NTP selection prior to the act of catalysis. By analogy with DNAPs and ssRNAPs, the existence of a conformational change in the active centre of multi-subunit RNAPs (TL folding) that brings the active centre to a catalytically active conformation suggests that substrate discrimination may take place via differential efficiency of conformational change occurrence upon the binding of cNTPs versus ncNTPs. In support of this idea, inhibition of TL folding of yeast RNAP II by α-amanitin resulted in a substantial decrease in the accuracy of NTP addition [10]. In contrast, the recent study on *E. coli *RNAP suggested that the open state of the active centre (with unfolded TL) may be a major checkpoint for discrimination against ncNTPs and c2'dNTPs [13].

Here, by using structure-based mutagenesis and fast kinetic analysis of phosphodiester bond formation by wild-type (WT) and mutant *T. aquaticus *RNAPs we uncover a stepwise mechanism of transcription fidelity, and dissect the role of TL in this process. Our results also reveal the role played by TL in the catalysis of phosphodiester bond formation.

## Results

### Experimental set up and kinetic analysis

In order to establish the roles of the open and closed states of the active centre in catalysis and fidelity, we have constructed RNAP lacking TL (ΔTL RNAP) [7] and several RNAPs bearing substitutions of TL amino acids that directly face/contact the cNTP bound in the i+1 site when TL is folded [9]. These amino acids included: Q1235, M1238, R1239, F1241, H1242 and T1243 (Figure 1). Compared to WT, F1241A and T1243A substitutions caused only minor, three- to fourfold defects in all reactions catalysed by the RNAP active centre (cNTP addition, pyrophosphorolysis, hydrolysis; see Additional File 1: Figure S1A, B, C, respectively). We considered these effects non-specific and did not investigate them further.

**Figure 2:**
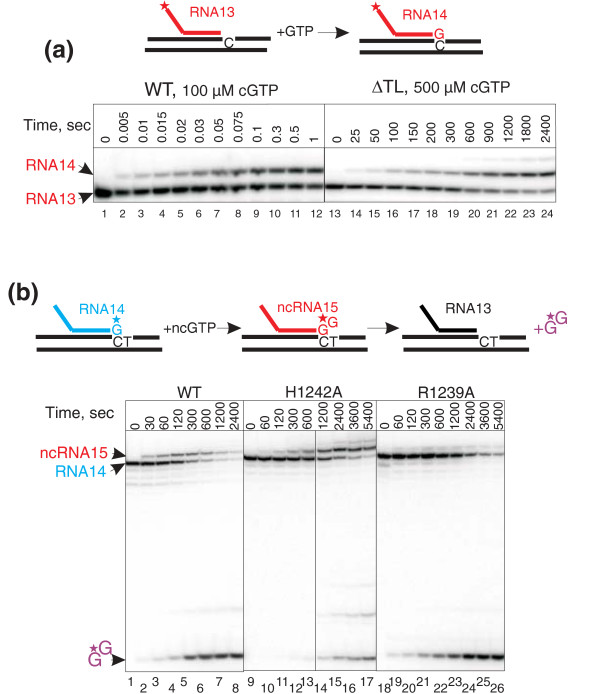
**Incorporation and misincorporation by wild-type (WT) and mutant RNA polymerase (RNAPs)**. (a) Cartoon schematically describes the reaction of cNTP (cGTP) incorporation in EC^G1 ^(Additional File1: Figure S2) with ^32^P 5'-labelled RNA (asterisk). Elongation complexes are shown with non-template DNA strand below the template strand to reflect their full complementarity (as in Additional File 1: Figure S2). Representative gels of 100 μM cGTP incorporation by WT and 500 μM cGTP incorporation by ΔTL in EC^G1 ^are shown. The lack of complete extension of transcripts was due to the procedure by which elongation complexes were assembled (see Methods). (b) The intrinsic proofreading reaction accompanies misincorporation. The cartoon above the gel schematically describes the processes going on during ncGTP misincorporation in EC^A ^(Additional File 1: Figure S2). Elongation complexes are shown with non-template DNA strand below the template strand to reflect their full complementarity (as in Additional File 1: Figure S2). Note that RNAs in elongation complexes were labelled at the 3' end (by incorporation of [α^32^P]GTP; asterisk), thus allowing monitoring both misincorporation event and removal of the wrong nucleotide via transcript assisted proofreading. Misincorporation of 1 mM ncGTP and proofreading by WT, H1242A and R1239A RNAPs are shown as an example. The cleavage products larger than dinucleotide originate from 2 bp and 3 bp backtracked complexes that undergone further extension after misincorporation. The colours of the RNA products of the reactions are the same as in the scheme of the reaction above the gels. Black vertical line separates lanes originating from the same gel that were brought together.

For the remaining mutants, we determined *k*_pol _(catalytic rate at saturating substrate concentration) and K_d _(substrate dissociation constant) of single nucleotide incorporation and misincorporation reactions using artificially assembled elongation complexes [7,15,16] (Figure 2, Additional File 1: Figure S2). The kinetic analysis of the data was performed as described in Materials and Methods and Additional File 1: Supplementary Methods.

Given that the rates of the reactions catalysed by RNAP can be influenced by the translocation state of the elongation complex (pre- versus post-translocated), we used elongation complexes in which the translocation state equilibriums were not significantly affected by RNAP mutations (Additional File 1: Figure S3).

We have previously shown that misincorporated ncNMPs are efficiently removed from nascent RNA through transcript-assisted hydrolysis of the second (with respect to the 3' end of the RNA) phosphodiester bond of the transcript [15]. Therefore, in order to measure the rate of misincorporation we needed to monitor both the extension of the nascent RNA with ncNMP and the subsequent transcript cleavage. In order to achieve this goal, initial transcripts were labelled at the 3' end allowing us to simultaneously observe single-nucleotide addition upon misincorporation and the generation of dinucleotide through transcript-assisted proofreading (Figure 2b). Note, for example, that, as seen from Figure 2b, it would have been virtually impossible to compare misincorporation by R1239A and H1242A RNAPs if only the RNA extension is monitored. Although they have similar rates of misincorporation, these RNAPs have very different rates of proofreading: the R1239A substitution does not influence intrinsic cleavage, while H1242A strongly diminishes it. This result also suggests that TL plays an important role in intrinsic RNA hydrolysis, and this is being investigated in a separate study.

### Active centre of ΔTL RNAP is stabilized in the open conformation

In order to investigate the role of TL in the active centre function, we constructed an RNAP mutant that lacked the entire TL (β' amino acids 1238-1254), ΔTL RNAP [7]. In accordance with previous results [7,9,13,17], ΔTL RNAP incorporated cNTP four orders of magnitude slower than WT RNAP (Figure 2a and Table 1), while the values of K_d _for cNTP remained largely unchanged (Table 1). The same defect in cNTP incorporation was observed for WT RNAP inhibited by the antibiotic streptolydigin (WT/Stl RNAP, see Table 2) [7], which locks the active centre in an open state by blocking the folding of TL [7,9]. Stl binds to but does not inhibit ΔTL RNAP, indicating that blocking of TL folding is the main pathway of Stl action [7]. Taken together, these data indicate that the active centre of ΔTL RNAP permanently remains in an open state, where the enzyme can accept incoming NTPs but cannot efficiently incorporate them into RNA. Comparison of the properties of WT and ΔTL RNAPs gave us an opportunity to distinguish between TL-dependent and TL-independent functions and to reveal the role played by TL in catalysis and fidelity.

**Table 1:** K_d _and *k*_pol _for incorporation and misincorporation by wild-type (WT) and ΔTL RNA polymerase (RNAP)

RNAP	WT		ΔTL	
	***k*_pol _(s^-1^)**	**K_d _(μM)**	***k*_pol _(s^-1^)**	**K_d _(μM)**

cGTP	100	20	1.6 ± 0.1 × 10^-3^	36 ± 6

ncGTP	2.7 ± 0.3 × 10^-2^	2800 ± 600	4.2 ± 0.5 × 10^-4^	840 ± 190

c2'dATP	5.5 ± 0.2 × 10^-2^	400 ± 30	1.3 ± 0.1 × 10^-3^	50 ± 9

c3'dATP	1.4 ± 0.1× 10^-1^	55 ± 8	2.7 ± 0.2 × 10^-3^	57 ± 11

Incorporation of cGTP was studied in EC^G1^, misincorporation of ncGTP, c2'dATP and c3'dATP - in EC^A^. K_d _and *k*_pol _(reaction rate at saturating nucleotide triphosphate concentration) for incorporation by WT RNAP were obtained by fitting the kinetics data into a simple kinetic model as described in Additional File 1:Supplementary Methods. The rest of the data were fitted into the Michaelis-Menten equation as described in Materials and Methods. Errors are the standard error of data fitting. The values were rounded to two significant digits.

**Table 2:** K_d _and *k*_pol _for incorporation and misincorporation by mutant RNA polymerase (RNAP)

	cGTP incorporation		ncGTP misincorporation	
**RNAP**	***k*_pol _(s^-1^)**	**K_d _(μM)**	***k*_pol _(s^-1^)**	**K_d _(μM)**

WT/Stl	3.6 ± 0.2 × 10^-3^	24 ± 6	1.1 ± 0.4 × 10^-4^	710 ± 70

H1242A	9.8 × 10^-1^	20	1.7 ± 0.2 × 10^-3^	2300 ± 500

R1239A	2.1	20	2.7 ± 0.3 × 10^-3^	2700 ± 500

H1242A/R1239A	6.9 ± 0.5 × 10^-2^	37 ± 11	4.0 ± 0.4 × 10^-4^	2900 ± 500

M1238A	5.6 ± 0.5 × 10^-2^	47 ± 17	3.6 ± 0.3 × 10^-4^	990 ± 230

Incorporation of cGTP was studied in EC^G1^, misincorporation of ncGTP - in EC^A^. K_d _and *k*_pol _(reaction rate at saturating nucleotide triphosphate concentration) for incorporation by R1239A and H1242A RNAPs were obtained by fitting the kinetics data into a simple kinetic model as described in Additional File 1: Supplementary Methods. K_d _and *k*_pol _for incorporation by other RNAPs and misincorporation by all RNAPs was obtained by fitting into the Michaelis-Menten equation as described in Materials and Methods. Errors are the standard error of data fitting. The values were rounded to two significant digits.

### Fidelity of RNAP active centre in the open state

In order to establish the role played by TL in discrimination against non-complementary substrates we analysed kinetic discrimination for all possible misincorporation events by ΔTL and WT RNAPs (Table 3). Most misincorporation reactions by ΔTL RNAP proceeded extremely slowly even in the presence of 10 mM ncNTP substrates, indicating that high (~500-5000-fold, see Table 3; low extension efficiency disallowed accurate quantification) levels of discrimination are achieved against these misincorporation events in the open active centre. Although further improved by TL, discrimination against these events predominantly takes place in the open active centre (Table 3). For simplicity we refer to these misincorporation events as unNTPs (for unusable). Some misincorporation events, however, were poorly discriminated against by ΔTL RNAP (~4-40-fold, bold in Table 3). For simplicity, we refer to these misincorporation events as ncNTPs (for non-complementary). The presence of TL significantly (in some cases dramatically) improved discrimination against ncNTPs. The most pronounced TL-dependency was observed for discrimination against ncGTP misincorporation in position coding for cATP (Table 3). We therefore focused on the examination of this misincorporation event as an example of TL-dependent discrimination, and refer to it as ncNTP throughout the text.

**Table 3:** Discrimination against various misincorporation events by ΔTL and wild-type RNA polymerase (WT RNAP).

ΔTL *WT*	ATP	CTP	GTP	UTP
EC^A^(cATP)	-	~1500 *20000*	**~4** ***~1200***	**~20** ***~3000***

EC^C^(cCTP)	~1000 *~100000*	-	~400 *~30000*	**~20** ***~350***

EC^G2^(cGTP)	**~40** **~*450***	~5000 *~60000*	-	**~20** ***~750***

EC^U^(cUTP)	~2000 *~100000*	~500 *~1000*	~900 *~65000*	-

Kinetic discrimination (folds) was quantified as a ratio of the rate of incorporation of 1 mM cATP, cCTP, cGTP and cUTP (measured in elongation complexes EC^A^, EC^C^, EC^G1 ^and EC^U^, respectively (Additional File 1: Figure S2)) to the rate of misincorporation of these 10 mM nucleotide triphosphates (top row) in elongation complexes identified in the left column (Additional File 1: Figure S2). The data for ΔTL RNAP (plain) are approximate due to very low efficiency of some of the misincorporation events by ΔTL RNAP. The data for WT RNAP (*Italic*) are approximate because incorporation was fitted using single exponent equation.

WT RNAP incorporated ncNTP three orders of magnitude slower than cNTP. The rate of misincorporation of ncNTP by ΔTL RNAP was only slightly slower than incorporation of the cNTP (Table 1). WT/Stl RNAP misincorporated ncNTP at a rate similar to that of ΔTL RNAP (Table 2). Given that the active centres of ΔTL and WT/Stl RNAPs are stabilized in the open state, we conclude that the open state does not allow efficient discrimination based on the kinetics of incorporation of cNTP versus ncNTP misincorporation. This important result was not influenced by the differences in the elongation complexes we used for incorporation and misincorporation analysis (Additional File 1: Supplementary Text).

### Induced fit discrimination and active expulsion of ncNTP from the active centre

As mentioned above, TL does not influence the affinity of the RNAP active centre for cNTP. We measured K_d _[ncNTP] for WT and ΔTL RNAPs. The affinity of ΔTL (and WT/Stl) RNAP for ncNTP was ~20 times lower than that for cNTP (Table 1). The difference in K_d _[cNTP] and K_d _[ncNTP] is probably due to improper hydrogen bonding with the template base in the case of ncNTP and is in agreement with theoretical calculations of base pair formation between non-cognate nucleotide bases [18]. Unexpectedly, K_d _[ncNTP] for WT RNAP was significantly higher than that for ΔTL (Table 1) or WT/Stl (Table 2) RNAPs, indicating that ncNTP bound in the i+1 site and the folded TL compete with each other. According to the structural data on *T. thermophilus *RNAP and yeast RNAP II, cNTP and folded TL are structurally complementary (Figure 1) [9,11]. The competition between folded TL and ncNTP can, therefore, be explained by the absence of such complementarity, probably caused by an altered geometry of a non Watson-Crick (G-T) base pair and, as a result, by a steric collision between them. Given that the folded TL participates in phosphodiester bond formation (see below), steric competition of ncNTP bound in the i+1 site with the folded TL leads to a diminished rate of phosphodiester bond synthesis - to a slow misincorporation. No such competition takes place in the case of cNTP bound in the active centre (Table 1), which allows efficient TL folding and catalysis. Therefore, TL-dependent cNTP incorporation proceeds much faster than TL-dependent ncNTP misincorporation, leading to kinetic discrimination against ncNTP.

The mainly kinetic (rather than affinity) based selection of ncNTPs by RNAP suggests that discrimination takes place via an induced fit mechanism [10]. From our results it follows that: (i) TL is required for catalysis (and participates in it, see below); and (ii) TL is sterically conflicting with the ncNTP bound in the active centre (as follows from their competition in the active centre). This suggests that the conformational change (TL closing) in the active centre required for incorporation of cNTPs is obstructed in the presence of ncNTPs, thus supporting the induced fit discrimination hypothesis. TL-dependent induced-fit kinetic discrimination, therefore, is another step assuring fidelity of transcription.

### M1238 governs TL folding and expulsion of ncNTP from the active centre

**Figure 3:**
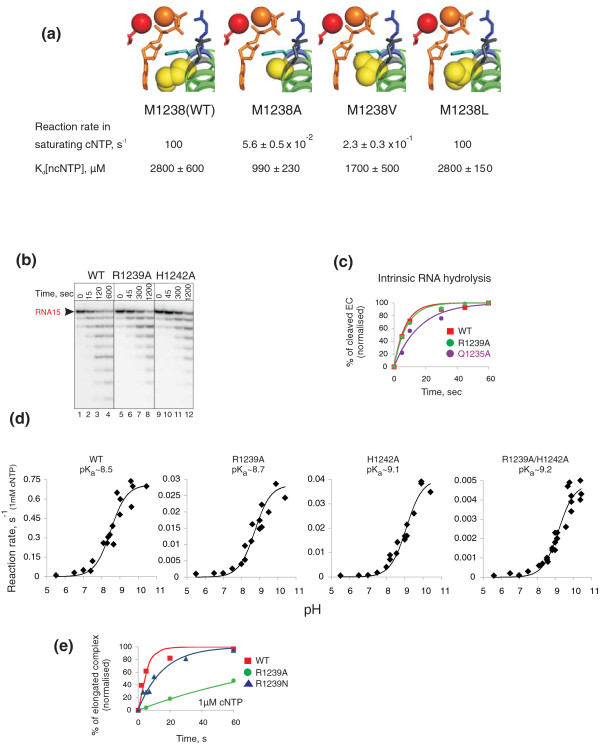
**Roles of TL amino acids in catalysis**. (a). Reaction rates in saturating (1 mM) cGTP in EC^G1 ^and K_d _[ncGTP] by wild-type (WT), M1238A, M1238V and M1238L are shown below the cartoons of the active centres of the corresponding enzymes, drawn in PyMol using PDB 2O5J and 'mutagenesis' function (colour code as in Figure 1). (b) Kinetics of pyrophosphorolysis by WT, R1239A and H1242A in the presence of 0.5 mM PP_i _in EC^G1 ^(Additional File 1: Figure S2) with ^32^P 5'-labelled RNA that was walked by two positions (G and A). (c) Kinetics of intrinsic transcript hydrolysis by WT (red squares), Q1235A (violet circles) and R1239A (green circles) RNA polymerase (RNAP) in EC^hydr ^(Additional File 1: Figure S2) with ^32^P 5'-labelled RNA. The lines in the plot are the non-linear regression fits of the data. (d) pH dependences of the rates of 1 mM cGTP incorporation in EC^G1 ^at 20°C by WT, R1239A, H1242A and R1239A/H1239A RNAPs. Solid lines show fits of the data to a sigmoidal function, and pK_a _values retrieved from these fits are shown above the plots. (e) Kinetics of 1 μM cGTP incorporation in EC^G1 ^by WT (red squares), R1239A (green circles) and R1239N (blue triangles). Solid lines show fits of the data to an exponential function.

In order to further investigate the competition between folded TL and ncNTP bound in the active centre, we analysed K_d _[ncNTP] for RNAPs bearing single substitutions in TL. From the mutant enzymes tested (Table 2) only M1238A RNAP had K_d _[ncNTP] that was significantly lower than the WT RNAP value and close to that of ΔTL RNAP (Table 1). This suggests that M1238 is required for ncNTP expulsion from the active centre. In the folded TL, M1238 stacks on the base of cNTP in the i+1 site (Figure 1, 3a). We hypothesized that M1238 is required for stabilization of the TL in the folded conformation via interaction with the incoming substrate base and, thus, is responsible for the observed competition between folded TL and ncNTP. To test this notion, we analysed RNAPs with substitutions of M1238 to hydrophobic amino acids with larger than alanine side chains, M1238V and M1238L (Figure 3a). In support of our hypothesis, M1238V RNAP had K_d _[ncNTP] = 1700 ± 500 μM (that is, intermediate between those of M1238A and WT RNAPs) while M1238L RNAP had the same K_d _[ncNTP] = 2800 ± 150 μM as WT RNAP (Figure 3a).

Importantly, the ability to expel ncNTP from the active centre by M1238A, M1238V, and M1238L RNAPs correlated with catalytic activities of these enzymes: in cNTP addition reaction, M1238V RNAP was slightly faster than M1238A, while M1238L was as fast as WT RNAP (Figure 3a). The correlation between catalytic activity and efficiency of competition of folded TL with ncNTP bound in the active centre suggests that 'TL folding required for catalysis' and 'TL folding required for ncNTP expulsion from the active centre' are one and the same process. Interestingly, in eukaryotic RNAPs II and III, the residue equivalent to M1238 of bacterial RNAP is leucine. Taken together, our data suggest that M1238 is required for stabilization of the folded state of the TL in the presence of correct substrate in the i+1 site. In the case of ncNTP, we propose that this stabilization leads to a steric competition between bound ncNTP and the folded TL and, as a result, to the removal of incorrect substrate from the active centre. The removal of erroneous NTP from the active centre upon TL folding contributes substantially to overall transcription fidelity (around an order of magnitude; Table 4), thus being another step assuring accurate transcription.

**Table 4:** Discrimination against 2' and 3'-deoxy substrates by ΔTL RNA polymerase.

	cATP(EC^A^)/ c2'dATP(EC^A^)	cCTP(EC^C^)/ c2'dCTP(EC^C^)	cGTP(EC^G3^)/ c2'dGTP(EC^G2^)	cUTP(EC^U^)/ TTP(EC^U^)	cATP(EC^A^)/ c3'dATP(EC^A^)	cGTP(EC^G3^)/ c3'dGTP(EC^G2^)
Discrimination, folds	~6	~11	~3	~17	~3	~0.4

Kinetic discrimination was quantified as a ratio of the rate of incorporation of 1 mM cNTP to the rate of misincorporation of 1 mM of a corresponding c2'dNTP or c3'dNTP in elongation complexes identified.

### Role of TL in catalysis

We further investigated the basis for the TL-dependent kinetic step of transcription fidelity. In the crystal structure, R1239 and H1242 of the folded TL contact phosphates of the triphosphate moiety of cNTP bound in the i+1 site [9] (Figure 1). Alanine substitutions of either R1239 or H1242 reduce the rate of cNTP incorporation 50-100-fold (Tables 1 and 2). The effects of these substitutions were cumulative: the double alanine substitution (R1239A/H1242A) led to a further decrease in the rate of cNTP incorporation (Table 2), which was approximately the multiplicative product of individual defects observed for R1239A and H1242A mutants. This indicates that R1239 and H1242 act at the same kinetic step of catalysis. Furthermore, the rate of pyrophosphorolysis was also affected similarly by R1239A and H1242A substitutions (Figure 3b, Additional File 1: Table S3), suggesting that R1239 and H1242 play functionally analogous roles during catalysis of phosphotransfer reactions. In order to test if the decreased rate of cNTP incorporation by mutant RNAPs is caused by distortion of TL folding, we analysed the ability of R1239A/H1242A TL to displace ncNTP from the active centre. As can be seen from Table 1, R1239A/H1242A RNAP has K_d _[ncNTP] similar to that of WT (and significantly higher than those of ΔTL or WT/Stl). Furthermore, R1239A substitution had no effect on intrinsic RNA hydrolytic activity (Figure 3c), which also requires TL folding [7]. These data indicate that TL with R1239A/H1242A double substitution has retained its ability to fold and that the defect caused by this mutation is caused by deficiency in catalysis *per se*. The magnitude of R1239A/H1242A substitution effect on phosphodiester bond formation indicates that these two amino acids are the main determinants of TL function in catalysis.

**Figure 4:**
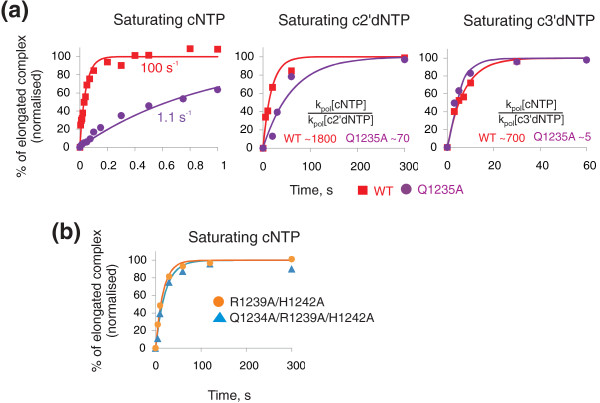
**Role of Q1235 in discrimination against c2'dNTPs and c3'dNTPs and in catalysis**. (a) Kinetics of incorporation of saturating cGTP (1 mM) in EC^G1^, c2'dATP (4 mM) and c3'dATP (1 mM) in EC^A ^by wild-type (WT; red squares) and Q1235A (violet circles) RNAPs. Kinetic discrimination against c2'dATP and c3'dATP was quantified as a ratio of the rate of cGTP incorporation to the rate of incorporation of corresponding erroneous substrate. (b) Q1235 does not participate in catalysis directly. Kinetics of saturating (1 mM) cGTP incorporation in EC^G1 ^by R1239A/H1242A (orange circles) and Q1235A/R1239A/H1242A (cyan triangles) RNA polymerase. Compare to panel A (left plot) and to Figure 3e.

The similarity of the effects caused by individual R1239A and H1242A substitutions on phosphotransfer reactions disfavours the exclusive role of H1242 in catalysis as a Brønsted-Lowry or Lewis acid [11,12]. Nevertheless, we measured pH dependences of catalytic rates of WT, R1239A, H1242A and R1239A/H1242A RNAPs. We used 1 mM cNTP to avoid effects of pH on K_d _[cNTP]. The shapes of curves for all four RNAPs were the same (Figure 4d). The descending limb that corresponds to protonation of the leaving group was not observed in either of the plots. Inflection at pH = ~8.5-9.2 can be assigned to pK_a _of deprotonation of RNA 3' hydroxyl - that is, of a general base. Note that the substitution of either R1239 or H1242 shifts this pK_a _towards a more basic value, though by less than one pH unit (Figure 4d). Therefore, H1242 and R1239 participate in general base catalysis (in contrast to earlier proposed role in general acid catalysis [12]), but are likely do so as a part of a network of other groups of the active centre. This view is supported by the fact that the slopes of log-log curves were less than 1 indicating that the inflection point does not reflect a single pK_a _that is being titrated. Furthermore, substitution of R1239 to asparagine (naturally present in this position in eukaryotic RNAP II) only slightly affected catalysis (Figure 4e), although the side chains of these amino acids are of different chemical nature.

Therefore, in agreement with crystallographic studies of *T. thermophilus *RNAP elongation complex [9], our results suggest that R1239 and H1242 coordinate the triphosphate moiety of cNTP in the i+1 site to allow efficient transition state formation (transition state stabilization and/or orientation catalysis), although they are also indirectly participating in general base catalysis. We therefore propose that the kinetic induced-fit discrimination against ncNTP is based on different efficiencies of transition state stabilization and/or reactants orientation and, in part, of base catalysis during cNTP incorporation versus ncNTP misincorporation.

### Discrimination against 2'-deoxyNTP

Incorporation of 2'-deoxyNTPs in RNA may be detrimental to cells as 2' hydroxyls are frequently involved in RNA ternary structure and function and may influence mRNA translation. We therefore investigated the mechanisms assuring discrimination against complementary 2'-deoxyNTPs (c2'dNTPs) during transcription. As can be seen from Table 1, ΔTL RNAP only poorly distinguished between c2'dNTP and cNTP, while discrimination by WT RNAP was strong (~2000 fold, see Tables 1, 4 and 5). The observed poor kinetic discrimination by ΔTL RNAP was not due to differences in the elongation complexes used for cNTP and c2'dNTP incorporation (Additional File 1: Supplementary Text). Low kinetic discrimination by ΔTL RNAP was observed for all c2'dNTPs (Table 4). The highest discrimination was achieved against cTTP, which was incorporated only 17 times slower than cUTP (Table 4). The result indicates that in the open state of the active centre, discrimination against c2'dNTP is ineffective; in other words, discrimination against c2'dNTP mainly depends on the presence of TL.

The kinetic discrimination against c2'dNTPs implies that the induced fit mechanism is involved [10]. We tested if TL folding, that apparently determines induced fit discrimination, would result in expulsion of c2'dNTP from the active centre - that is, if K_d _[c2'dNTP] of WT RNAP would be higher than that of ΔTL RNAP. As can be seen from Table 1, WT RNAP indeed had ~10-fold higher K_d _[c2'dNTP] than ΔTL RNAP, indicating that TL competes with c2'dNTP in the i+1 site. The competition between c2'dNTP and TL leads to the inhibition of TL folding and, thus, of productive catalysis compared to cNTP which does not compete with TL. The result supports the idea that discrimination against c2'dNTP proceeds via induced fit mechanism. The result also suggests that, as in the case of ncNTPs, TL participates in the active removal of c2'dNTP from the active centre.

**Figure 5:**
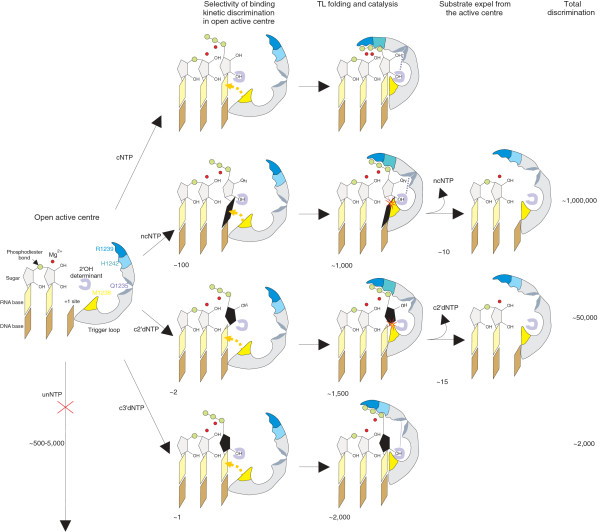
**The stepwise mechanism of transcription fidelity**. Cartoon schematically shows RNA polymerase active centre and the steps of discrimination against various erroneous substrates. From top to bottom: incorporation of complementary nucleotide triphosphate (cNTP), misincorporation of ncNTP, c2'dNTP and c3'dNTP. Discrimination against unNTP (unusable substrates) takes place in the open state of the active centre and is not shown. The approximate discrimination contributions, found in our study, are shown for each step.

It is likely that the absence of an interaction of the 2'OH with some determinant in the active centre makes c2'dNTP adopt a conformation that results in competition with the folded TL and the expulsion of c2'dNTP. We tested two amino acids, β' N737 and Q1235, that were earlier suggested to participate in the discrimination against c2'dNTP based on crystallographic and biochemical data with bacterial RNAPs and yeast RNAP II [9,11,19]. The substitution N737A had a small effect on c2'dNTP discrimination (Additional File 1: Table S1). In contrast, the substitution Q1235A led to a ~25-fold decrease in discrimination against c2'dNTP (Figure 4a). The result suggests that Q1235 participates in recognition of the 2'OH of cNTPs, but does it along with some other determinants. Q1235 may be partnered by β'R704 which, in the crystal structures of *T. thermophilus *RNAP and yeast RNAP II elongation complexes, is close to the 2'OH of the substrate [9,11]. The contributions of different steps in discrimination against c2'dNTP are summarized in Table 5 and are schematically shown in Figure 5.

**Table 5:** Contribution of individual fidelity steps.

Fidelity contribution	Open active centre	Trigger loop folding	Total
(*k*_pol _[cGTP]/K_d _[cGTP])/(*k*_pol _[ncGTP]/K_d _[ncGTP])	87	5950	518000

Kinetic *k*_pol _[cGTP]/*k*_pol _[ncGTP]	3.8	970	3700

Affinity K_d _[ncGTP]/K_d _[cGTP]	23	6.1	140

(*k*_pol _[cGTP]/K_d _[cGTP])/(*k*_pol _[c2'dATP]/K_d _[c2'dATP])	1.7	21000	36000

Kinetic *k*_pol _[cGTP]/*k*_pol _[c2'dATP]	1.2	1500	1800

Affinity K_d _[c2'dATP]/K_d _[cGTP]	1.4	14	20

(*k*_pol _[cGTP]/K_d _[cGTP])/(*k*_pol _[c3'dATP]/K_d _[c3'dATP])	0.96	2030	1990

Kinetic *k*_pol _[cGTP]/*k*_pol _[c3'dATP]	0.6	1180	710

Affinity K_d _[c3'dATP]/K_d _[cGTP]	1.6	1.8	2.8

Values were quantified using data of Table 1: 'Open active centre' values were generated from the row identifier ratios using the ΔTL data; 'Total' values were generated from the row identifier ratios using the wild-type (WT) data; 'TL folding' values were generated from the row identifier ratios using the ratio of the WT ratio over the ΔTL ratio.

### Discrimination against 3'-deoxyNTP

Incorporation of NTPs bearing modifications of the 3' hydroxyl or lacking this group abolishes further transcription. We therefore analysed the mechanism(s) used by RNAP to discriminate against complementary 3'-deoxyNTPs (c3'dNTPs). WT RNAP incorporated c3'dNTP ~700 times slower than cNTP (Table 1). In contrast, ΔTL RNAP practically failed to discriminate against c3'dNTP (Tables 1, 4 and 5), suggesting that discrimination strictly depends on the presence of TL. The phenomenon was not due to the differences in elongation complexes or the identity of c3'dNTP (Additional File 1: Supplementary Text). Next, we measured K_d _[c3'dNTP] for WT and ΔTL RNAPs. Surprisingly, WT RNAP had the same K_d _[c3'dNTP] as ΔTL RNAP (Table 1), indicating that c3'dNTP does not compete with, and is therefore not expelled by, the folded TL. Therefore, only kinetic TL-dependent discrimination is utilized against c3'dNTPs.

We investigated the mechanism of TL-dependent kinetic discrimination against c3'dNTP. Based on crystallographic studies of yeast RNAP II elongation complexes, it was suggested that Q1235 of TL and β'N737 may act in concert to check for the presence of the 3' OH [11]. We tested RNAP bearing Q1235A substitution for its ability to discriminate against c3'dNTP. As can be seen from Figure 4a, Q1235A RNAP showed only ~fivefold discrimination against c3'dNTP, compared to ~700-fold discrimination exhibited by WT RNAP. Therefore, Q1235 of TL is the primary determinant of kinetic discrimination against c3'dNTP. In line with this idea, N737A RNAP had a slight (fivefold) decrease in c3'dNTP discrimination which was, however, TL-dependent, because in the absence of TL, N737A substitution had no effect (ΔTL/N737A RNAP) (Additional File 1: Table S1).

We investigated the mechanism of Q1235-dependent kinetic discrimination. Q1235A substitution slowed down cNTP incorporation ~100 fold as compared to WT (Figure 4a). However, this effect was not due to altered TL folding as inferred from K_d _[ncNTP] (4 ± 1 mM). Furthermore, the Q1235A substitution did not significantly influence intrinsic transcript hydrolysis (Figure 3c), which also requires TL folding [7]. In order to test if Q1235 participates, along with R1239 and H1242, in catalysis, we analysed Q1235A/R1239A/H1242A triple mutant RNAP. Curiously, while the single Q1235A substitution had a significant effect on the rate of reaction, the triple mutant did not have any further defect in catalysis than the double R1239/H1242 mutant (Figure 4b). The result suggests that Q1235 does not directly participate in catalysis along with R1239 and H1242 but is somehow linked to the function of these amino acids, possibly acting at the step preceding transition state stabilization by R1239/H1242. Note also that Q1235A and R1239A substitutions have similar effects on catalysis and, in the crystal structure of yeast RNAP II elongation complex, Q1078 (a homologue of Q1235 of *T. aquaticus *RNAP) interacts with both N1082 (a homologue of R1239 of *T. aquaticus *RNAP) and the 3'OH of the substrate [11]. These data suggest that Q1235 may participate in orientation of R1239 into a catalytically active conformation. When the incoming NTP lacks the 3' OH, Q1235 fails to perform this role, leading to inefficient catalysis and inefficient kinetic discrimination against c3'dNTPs. The contributions of different steps in discrimination against c3'dNTP are summarized in Table 5 and are also shown schematically in Figure 5.

## Discussion

Taken together, our results suggest that the fidelity of transcription is achieved via a stepwise process and that each step contributes differently to discrimination against non-complementary, 2'-deoxy, and 3'-deoxy erroneous substrates. The scheme summarizing our findings is presented in Figure 5. These steps are based on two conformational states of the RNAP active centre (open and close) and on a structural rearrangement of the active centre during transition between these two states. The open active centre can efficiently discriminate only against unNTPs (which, apparently, cannot base pair with template DNA) but is error-prone in the case of ncNTPs, c2'dNTP and c3'dNTPs. Similar conclusions were made during analysis of yeast RNAP II [10]. That a 'motionless' active centre (that is, which lacks additional conformational changes) is error-prone appears to be a general concept, as illustrated, for example, by primases, which have error rates similar to that of ΔTL RNAP [20,21].

The poor fidelity achieved in the open state of the RNAP active centre does not have a strong influence on the accuracy of transcription because this state is catalytically very inefficient. Folding of TL that makes the active centre catalytically efficient therefore determines the following steps that contribute to transcription fidelity. Our results indicate that the catalytic role of TL is primarily determined by R1239 and H1242, which stabilize reaction transition state (or orient the reactants) and also indirectly contribute to general base catalysis. Thus, folding of TL, which is governed by M1238, is mainly required in order to bring R1239 and H1242 to the position from which they can accomplish this function. The proposed steric competition between folded TL and ncNTP or c2'dNTP bound in i+1 site makes catalysis with these incorrect nucleotides inefficient. There is no such competition between folded TL and cNTP. Therefore, though the rate of misincorporation is increased during TL folding (compared to the open state of the active centre), the increase of cNTP incorporation rate is much higher, leading to kinetic discrimination against ncNTPs and c2'dNTPs. Interestingly, in the case of c3'dNTP, TL is proposed to fold but fails to properly orient its catalytic residues in the absence of the functional relationship between Q1235 and the 3'OH.

The TL-controlled fidelity checkpoint is an example of an induced fit mechanism of enzymatic accuracy. DNAPs and single-subunit RNAPs also use an induced fit mechanism to ensure fidelity. In these enzymes, the binding of correct substrate induces a large conformational change converting the complex to a catalytically active, closed, conformation [22,23] (see also Introduction).

The principal finding of our study is that the apparent competition of the folded TL with ncNTP and c2'dNTP bound in the i+1 site results in the erroneous substrates' displacement from the active centre, further improving the fidelity by another order of magnitude (Table 5, Figure 5). The contribution of this step to transcription fidelity may be more important for c2'dNTPs, because these nucleotides bind in the active site with the same affinity as cNTPs and, therefore, should compete with correct substrates much more efficiently than ncNTPs. However, it should be noted that this step has no effect on the discrimination against c3'dNTPs.

Misincorporation rates by *T. aquaticus *WT RNAP measured in our study (Table 3) were qualitatively similar to those observed for yeast WT RNAP II [24], with two exceptions. *T. aquaticus *WT RNAP misincorporated ncGTP in place of cATP and ncUTP in place of cCTP much faster (relative to other misincorporation events) than did yeast WT RNAP II. This suggests that discrimination against some non-cognate NTPs may depend on the sequence of elongation complex or may vary between bacterial and eukaryotic RNAPs.

Curiously, results from a recent study on *E. coli *RNAP contradict some of our conclusions [13]. By studying *E. coli *ΔTL RNAP, the authors concluded that the open state of the active centre is able to efficiently discriminate against ncNTP. Although our conclusions agree on some misincorporation events (unNTPs: ncCTP/cATP and ncUTP/cATP), there is a discrepancy in the case of ncGTP/cATP misincorporation. Zhang *et al*. [13] argue that they failed to observe meaningful misincorporation at 10 mM ncGTP, while *T. aquaticus *RNAP misincorporated only six times slower than cNTP (Table 3, Additional File 1: Figure S5B). The elongation complexes used in both studies were of the same sequence. This factor can, therefore, be excluded when analysing the possible source of this discrepancy. We have constructed an *E. coli *ΔTL RNAP with a TL deletion identical to the one used by Zhang *et al*. [13] and tested it for ncGTP misincorporation (Additional File 1: Figure S5). We observed efficient misincorporation even in 1 mM ncGTP by the mutant enzyme, which was ~50 times slower than incorporation rate in the presence of 1 mM cGTP. Taking into account the higher K_d _[ncNTP] value, ~25-fold kinetic discrimination is expected in the open active centre, which is slightly higher than that observed for *T. aquaticus *ΔTL RNAP for the same misincorporation event. We also observed higher kinetic discrimination against c2'dNTP and c3'dNTP by *E. coli *ΔTL RNAP compared to *T. aquaticus *ΔTL RNAP in the same elongation complexes (Additional File 1: Figure S5).

Better discrimination by open-state active centre of *E. coli *RNAP is achieved due to the relatively faster TL-independent incorporation of cNTP by *E. coli *ΔTL RNAP compared to *T. aquaticus *ΔTL RNAP (compare Additional File 1: Figure S5, Table 1 and Figures 4 and 5 in [13]). This suggests that some amino acids of the *E. coli *RNAP active centre that lie outside TL may support catalysis and, thus, contribute to kinetic discrimination against erroneous NTPs in the open state of the active centre. This idea is supported by the fact that alanine substitutions of *E. coli *homologues of R1239 and H1242 have a far less drastic effect on catalysis (a four- to sixfold decrease) than the corresponding *T. aquaticus *RNAP substitutions (50-to 100-fold decrease; Tables 2). Therefore, *E. coli *possesses some additional catalytic mechanism that is lacking or diminished in *T. aquaticus *RNAP. Our results indicate that, in addition to TL-dependent transition state stabilization, the active centre performs general base catalysis which only partly depends on TL. It is therefore possible that *E. coli *RNAP relies on TL-independent base catalysis more than *T. aquaticus *RNAP and that this determines better kinetic discrimination in the open active centre. The drastic catalytic effect of the TL deletion in *E. coli *RNAP, however, indicates that this additional catalytic mechanism is still dependent on the TL, possibly through allosteric action of TL on amino acids of the active centre. Overall, our results suggest that the low accuracy of the open state of the active centre (except for unNTPs) is a general feature of multi-subunit RNAPs, though some species-specific variations are possible.

According to our results, the total fidelity of transcription that can be achieved through steps dissected in our study is ~10^-6 ^for ncNTPs, ~10^-5 ^for c2'dNTPs and ~10^-3 ^for c3'dNTPs (Tables 3, 4, 5). At high NTP concentrations present in cells, accuracy is expected to drop by two orders of magnitude in the case of ncNTPs and one order of magnitude for c2'dNTPs, but to remain the same for c3'dNTPs. However, the fidelity of transcription can be further improved by a factor of 10^-1^-10^-2 ^during the proofreading of transcription via transcript-assisted and cleavage factor-assisted (Gre in bacteria, TFIIS in eukaryotes and TFS in archaea) removal of erroneous nucleotides [15,25-27].

## Conclusions

Transcription is characterized by high fidelity of copying of genetic information into RNA. Discrimination against non-cognate and 2'- and 3'-deoxy NTPs entering the RNAP active centre proceeds via a stepwise mechanism involving not only discrimination based on Watson-Crick pairing but also active recognition of substrates by the catalytic domain of the RNAP active centre, the TL. Each step contributes differently to the overall fidelity against various erroneous substrates. A unique property of the multi-subunit RNAP active centre, compared to single-subunit RNAPs and DNAPs active centres, is the ability of the former to actively expel wrong substrates upon TL folding. This property may be particularly important in the case of 2'-deoxy NTPs, which are not discriminated during initial binding and, therefore, compete with the cognate rNTPs.

Induced fit discrimination based on kinetics of incorporation versus misincorporation is determined by the direct involvement of TL in catalysis along with the two Mg^2+ ^ions of the RNAP active centre. Interestingly, such combinatorial way of catalysis (two Mg^2+ ^ion catalysis + TL dependent transition state stabilization/base catalysis) indicates that the regulation of the activity of the enzyme can be accomplished through controlling TL folding, rather than the orientation of the catalytic Mg^2+ ^ions. These effects were observed during analysis of RNAPs with mutations in non-catalytic amino acids surrounding the active site [5,6,10,14,28]. Regulation of catalysis via TL may also be accomplished by external factors [29]. 'Combinatorial' catalysis also suggests that RNAPs of different organisms can adapt to various conditions by changing the amino acid composition of TL and surrounding amino acids, while leaving the two Mg^2+ ^binding sites virtually intact [1]. For example, changes in the F-loop, a domain close to TL, determine the temperature optima of catalysis by closely related RNAPs from thermophilic *Thermus aquaticus *and mesophilic *Deinococcus radiodurans *[30].

## Methods

### Mutant RNAP construction and purification

*T. aquaticus *RNAP lacking TL (β' residues 1238-1254 replaced with a glycine residue) was constructed as described [7]. Single alanine substitutions in the TL of *T. aquaticus *RNAP (β' M1238A, R1239A, F1241A, H1242A and T1243A) were obtained by the site directed mutagenesis in a co-expression system for recombinant RNAP [31]. WT and mutant core RNA polymerases were purified as described [31]. In order to obtain *E. coli *ΔTL RNAP (having β' residues 931-1137 replaced with three alanine residues [13]) the deletion was introduced in the β' subunit coded under inducible promoter in plasmid pRL663. Mutant RNAP production was performed as described [32] and its purification as described [33].

### Transcription assays

Elongation complexes (Additional File 1: Figure S2) [3,16] were assembled and immobilized on Ni-NTA beads (Qiagen, Venlo, The Netherlands) as described [15,16]. The beads were washed with 1 M KCl to remove incorrectly assembled complexes. For fast kinetics experiments the immobilization step was omitted (which results in incomplete incorporation, see Figure 2a). The presence of non-template strand in all complexes was confirmed by treatment with Exo III (digests only double stranded DNA, not shown). When specified, RNAP was walked to a desired position by the addition of required substrates with their subsequent removal through beads washing. RNA was either ^32^P labelled at the 5'end [15] or labelled at 3'-end after elongation complex assembly by incorporation of [α-^32^P]GTP (PerkinElmer, MA, USA) with subsequent removal of unincorporated [α-^32^P]GTP through beads washing. Transcription buffer contained 40 mM KCl and 20 mM Tris pH 8.0, unless otherwise specified. Intrinsic RNA hydrolysis was initiated by the addition of 10 mM MgCl_2_. Incorporation and pyrophosphorolysis reactions were initiated by the addition of a mixture 10 mM MgCl_2 _with NTPs or PP_i_, respectively. For misincorporation, 20 mM MgCl_2 _was used in order to avoid possible titrating out of the Mg^2+ ^by high NTP concentrations. Reactions were incubated at 40°C (20°C for pH dependences), stopped by the addition of 1 M HCl (final concentration) or formamide-containing buffer, neutralized if required and analysed by Urea-PAGE and Phosphorimaging (GE Healthcare, Buckinghamshire, UK). Exo III footprinting and hydroxyl radical probing of Additional File 1: Figure S3 was performed as described in the figure legend.

### Fast flow kinetics

Fast kinetic experiments were performed using a BioLogic Quenched-Flow QFM-400 instrument (Bio-logic SAS, Claix, France) equipped with 3.5 μL ageing line. The settings and calibration of the QFM-400 were accomplished according to the standard procedure suggested by the manufacturer. Experiments were conducted at 40°C (20°C for pH dependences) by using a circulating water bath. Assembled elongation complexes were loaded in the syringe 1 and a solution containing various concentrations of correct nucleotide and 10 mM MgCl_2 _were loaded in syringe 2. 30 μL samples from syringes 1 and 2 were mixed and the incorporation was allowed to proceed for the indicated times. Reactions were quenched by addition of HCl to a final concentration of 1 M from syringe 4. Immediately after the addition of HCl, the solution was neutralized by the addition of 1 M KOH and 300 mM Tris base (final concentration). Products were analysed as described above.

### Data analysis

For kinetics that are described by a single exponent (for other case see Additional File 1: Supplementary Information), we used *P *= *A *× (1-*e*^kt^), where *P *is the fraction of incorporated RNA, *A *is the fraction of active RNAP complexes, k is the reaction rate and t is the time. The rates obtained for various concentrations were fitted to the Michaelis-Menten equation; k = *k*_pol _× [NTP]/(K_M_^obs^+ [NTP]), where [NTP] is the substrate concentration, *k*_pol _is the maximum rate of the enzyme and K_M_^obs ^is the Michaelis constant. Prism 5 (GraphPad Software, CA, USA) was used to perform the single exponential fits to misincorporation and incorporation data. The kinetic simulations were performed using the ISRES algorithm [34,35] implemented in MATLAB (The MathWorks, MA, USA).). Nonlinear regression with least squares was used to obtain the best fit of the model to the data. We minimized the sum of squares between the experimental data and the data predicted by the model for a particular set of parameters  to obtain the best fit parameters.

## Abbreviations

DNAP: DNA polymerase; NTP: nucleotide triphosphate; RNAP: RNA polymerase; Stl: streptolydigin; ssRNAP: single-subunit RNAP; TL: Trigger loop; cNTP: complementary NTP; unNTP: unusable NTP; ncNTP: non-complementary NTP; WT: wild-type.

## Authors' contributions

AB carried out Quench Flow experiments. MR measured pH dependences. KS participated in designing pilot experiments and in drafting the manuscript. NZ designed and coordinated the study, analysed the data and wrote the manuscript. SZ participated in pilot experiments. VRT built the mathematical model, analysed the data and participated in drafting the manuscript. YY carried out experiments and analysed the data. All authors read and approved the final manuscript.

## Supplementary Material

Additional file 1 **Supplementary Information**. Contains supplementary text, supplementary methods, and supplementary figures and figure legends. Click here for file
