# Supplementary material for: Stepwise mechanism for transcription fidelity
Source: BMC Biol. 2010 May 7;8:54. doi: 10.1186/1741-7007-8-54 (PMC2874521; doi:10.1186/1741-7007-8-54)
Supplement: Additional file 1 — Supplementary Information. Contains supplementary text, supplementary methods, and supplementary figures and figure legends. [file 1741-7007-8-54-S1.pdf]

SUPPLEMENTARY INFORMATION

SUPPLEMENTARY TEXT

Though elongation complexes are expected to be uniform (except for pause sequences) in catalytic properties, the differences in elongation complexes used for measurements of incorporation of cGTP (EC^{G1}; Supplementary Fig. S2) and misincorporation of ncGTP (EC^{A}; Supplementary Fig. S2) may have influenced our conclusion about the inaccuracy of the open state of RNAP active centre. We therefore analysed incorporation of cGTP by ΔTL RNAP in elongation complex that was identical to EC^{A} (used for measuring misincorporation) except for i+1 position of DNA coding for GTP instead of ATP (EC^{G3} in Supplementary Fig. S2). As seen from Supplementary Fig. S4, the rate of cGTP incorporation in this complex was just slightly faster than in elongation complex we routinely used to measure incorporation (2.7×10^{-3} s^{-1} in EC^{G3} versus 1.6×10^{-3} s^{-1} in EC^{A}), indicating that indeed only low kinetic discrimination (4-6 fold) is achieved in the open active centre, and that our conclusions are valid.

To test if our conclusions about poor discrimination against c2'dATP and c3'dATP in the open active centre are influenced by the differences in elongation complexes used, we measured cATP incorporation by ΔTL in EC^{A} (the same elongation complex in which c2'dATP and c3'dATP incorporations were measured, Supplementary Fig. S2). The rate of saturating cATP incorporation in EC^{A} was 8.1×10^{-3} s^{-1}, approximately 5 times faster than that of cGTP incorporation in EC^{G1} (Supplementary Fig. S4). Therefore, only minor discrimination against c2'dNTPs and c3'dNTPs (~6 and ~3 fold) takes place in the open active centre, supporting our conclusions. The rate of cATP incorporation in EC^{A} by WT RNAP was also faster than that of cGTP in EC^{G1} (not shown), indicating that our conclusions about the role of TL in discrimination against c2'dNTPs and c3'dNTPs are valid.

SUPPLEMENTARY METHODS

Kinetic data analysis

In our study we compared k_{pol} (catalytic rate at saturating substrate concentration) and K_d (substrate dissociation constant) of substrates for WT and mutant RNAPs. Kinetics of incorporation by some of the mutant enzymes, and misincorporation by all RNAPs used in our study are described well with the single exponential kinetics. We therefore used Michaelis–Menten model to determine k_{pol}, and K_M^{obs} (apparent Michaelis constant) (see Materials and Methods of the main text). The K_M^{obs} for these situations can be equated to K_d, given that the catalytic rates were very slow, and K_M^{obs} were not changed by mutations. We therefore use K_d throughout the text to describe the affinity of RNAPs to substrates.

For fast RNAPs (such as WT, R1239, H1242A RNAPs) the kinetics of cNTP incorporation were clearly double exponential, indicating that there are two rate limiting steps during phosphodiester bond formation. This precluded us from using Michaelis–Menten model to fit the data. Therefore, to retrieve k_{pol} and K_d values for these RNAPs, we built a simple kinetic model consisting of three reversible steps: (i) oscillation between pre- and post-translocated states; (ii) reversible binding of NTP in the i+1 site; (iii) reversible synthesis of the phosphodiester bond (Supplementary Fig. S6A). This model is consistent with the current understanding of the RNAP function. The reversible phosphodiester bond synthesis was used because we observed high rates of pyrophosphorolysis from the product complex (see below).

We used short time scales in the modelling of incorporation, since the reaction goes to completion in ~ 1 second for higher NTP concentrations. This results in some of the lower NTP concentration curves not approaching the same level of incorporation as those of high NTP concentrations (due to presence of reverse reaction in the model). This, however, does not imply that further NTP addition is completely inhibited at low NTP concentrations, and

we observe reactions in all NTP concentrations approaching the same level of incorporation when the reactions are followed for longer times (not shown). This is consistent with slow rate of PPi release observed in single NTP addition experiments [1], and further justifies the usage of a reversible phosphodiester bond synthesis step in the model. Short time scales used allowed us to omit the pyrophosphate release step in the model.

We used K_M^{obs} (apparent Michaelis constant from single exponent fits; Supplementary Table S2) values as an upper bound for K_d in our kinetic simulations since in a single substrate case K_d cannot exceed K_M^{obs}. We constrain parameters in the kinetic model fits so that they make biological sense (e.g. we do not allow extremely high values for k_{pol} (<100 s^{-1})). 5000 was used as an upper bound for all other parameters apart from k^- in fitting misincorporation data. All concentrations in a data set are fit by a single set of parameters. We also reduce the parameter space by constraining the rates of the single amino acid mutants such that the translocation rates are the same as WT. It has been shown that biological models are universally sloppy [2, 3], i.e. there are multiple sets of parameters even in the restricted parameter space we are looking at that fit the data equally well. We focus mainly on the predictions of the model rather than the actual values of the parameters. In such a regime error ranges on the parameters are irrelevant.

The initial kinetic rates of the model were obtained from a global fit of WT incorporation data (Supplementary Fig. S6B and Supplementary Table S3). These rates were used as a starting point to fit incorporation data of mutant RNAPs. In agreement with our conclusions that R1239 and H1242 stabilise the transition state during phosphodiester bond synthesis, the data for H1242A and R1239A RNAPs was fitted into the model by changing only the catalytic rates: catalysis (k_{cat}) and pyrophosphorolysis (k_{ppi}) rates were different from those of WT, while the translocation rates and the K_d being the same (Supplementary Fig.

S6C, D, Supplementary Table S3). ΔTL data was also fitted in the model without significant changes in K_d or translocation rates (Supplementary Table S3).

The model predicted an unusually high rate of pyrophosphorolysis by WT RNAP from the product complex (P in Supplementary Fig. S6A). Our data on pyrophosphorolysis fully support this prediction (Supplementary Fig. S6H). The results on pyrophosphorolysis by H1242A and R1239A RNAPs (Fig. 3B) are also in good agreement with model predictions (Supplementary Table S3).

Misincorporation kinetics data of WT and various mutants are also well fit by the model (Supplementary Fig. S6). The removal of the ncNTP from the active centre by the TL folding was incorporated into NTP dissociation rate (k^-). As seen from Supplementary Table S3, the difference in the catalysis rate was sufficient to explain the misincorporation data for WT and mutant RNAPs (pyrophosporolysis was extremely slow with ncNTP and k_{ppi} was set to zero). All the incorporation and misincorporation data fit to the model with an RMSE < 0.04.

SUPPLEMENTARY FIGURE LEGENDS

Supplementary Figure S1 Mutations of F1241 and T1243 have minor effects on all activities of RNAP. a. Kinetics of 1 μM GTP incorporation in EC^{G1} (Supplementary Fig. S2) by WT, F1241A and T1243A. **b**. Kinetics of pyrophosphorolysis by WT, F1241A and T1243A in the presence of 0.5 mM PP_i in EC^{G1} (Supplementary Fig. S2) that was walked by two positions (G and A). **c**. Kinetics of intrinsic hydrolysis by WT, F1241A and T1243A in EC^{hydr} (Supplementary Fig. S2).

Supplementary Figure S2 Elongation complexes used in the study. The sequences of elongation complexes used in our study are shown. The non-template strand is shown below the template to reflect their full complementarity (as in Fig. 2).

Supplementary Figure S3 Mutations of the TL do not significantly influence translocation equilibrium. A. Exonuclease III (ExoIII) footprinting of the front edge of elongation complexes formed by WT and ΔTL RNAPs. Complexes were formed on EC^{G1} (Supplementary Fig. S2), with the only exception that it had 30bp longer downstream DNA [4], with ^{32}P 5'-labelled RNA and non-template DNA strand. Where indicated 1 mM GTP or its non-hydrolysable analogue (GPcPP) were added for indicated times before addition of ExoIII. ExoIII footprinting was performed essentially as described [4]. One unit of ExoIII (New England Biolabs) was added to each 15 μl reaction, followed by one minute incubation at 40°C before addition of stop solution. The DNA footprint and RNA images from the same gel are shown in top and bottom panels, respectively. **B**. Hydroxyl radical footprint of RNA in elongation complexes formed by WT and ΔTL. Hydroxyl radicals are generated by the Fe^{3+} ion immobilised in the RNAP active centre instead of Mg^{2+} as a result of Fenton reaction. Hydroxyl radicals cleave the adjacent RNA thus revealing relative translocation state of elongation complex. The footprinting was performed in EC^{G1} (Supplementary Fig. S2) with ^{32}P 5'-labelled RNA. The elongation complexes without $MgCl_2$ were supplemented with 100 μM $Fe(NH_4)_2(SO_4)_2$ and 10 mM DTT were indicated. The reaction was stopped and products were analysed as described in Materials and Methods for transcription reactions. The reactions were incubated for the times indicated. As a size control a ladder obtained by RNA pyrophosphorolysis from the same elongation complex was loaded (lane 9). Note that the bands generated by hydroxyl radical cleavage migrate between the pyrophosphorolysis bands due to the presence of 3' phosphate group in the former. The hydroxyl radical cleavage

therefore proceeds at the ultimate phosphodiester bond.

Supplementary Figure S4 The rates of incorporation of saturating cNTP in EC^{G1}, EC^{G3} and EC^{A} by ΔTL RNAP are similar.

Supplementary Figure S5 Discrimination against ncNTP, c2'dNTP and c3'dNTP by *E.coli* ΔTL RNAP. A. Kinetics of misincorporation of 1 mM GTP in EC^{A} (Supplementary Fig. S2) by *E.coli* ΔTL RNAP. Black vertical line separates lanes originating from the same gel that were brought together. **B**. The rates of misincorporation and discrimination against erroneous substrates in the elongation complexes designated at the left by *E.coli* ΔTL RNAP. For comparison discrimination by *T.aquaticus* ΔTL RNAP against corresponding substrates in the same elongation complexes are shown at the right.

Supplementary Figure S6 Simple kinetic model fits both incorporation and misincorporation data for WT and mutant RNAPs. A. Schematic of the kinetic model: (i) in the absence of NTP, the RNAP oscillates between the pre- and post-translocated states; (ii) NTP binds in the post-translocated state; (iii) the active site catalyses phosphodiester bond formation extending the transcript by one. The reverse reaction, pyrophosphorolysis also occurs, but at a slower rate. The parameters specifying the fitting are shown in Supplementary Table S3. **B**. Model fit to WT incorporation data: 5 μM (red), 10 μM (blue), 20 μM (light green), 50 μM (magenta), 100 μM (cyan), 250 μM (black) and 500 μM (dark green). **C.** Model fit to R1239A RNAP incorporation of 10 μM (red), 20 μM (blue), 50 μM (light green), 100 μM (black), 250 μM (magenta) and 500 μM (dark green) NTP. **D.** Model fit to H1242A RNAP incorporation of 20 μM (red), 50 μM (blue), 100 μM (light green), 250

μM (magenta) and 500 μM (black) NTP. **E.** Model fit to WT misincorporation data: 250 μM (red), 500 μM (blue), 750 μM (light green), 1000 μM (magenta), 1500 μM (black), 2000 μM (dark green), 2500 μM (cyan). **G.** Model fit to R1239A misincorporation of 500 μM (red), 750 μM (blue), 1000 μM (black), 1500 μM (light green), 2000 μM (magenta), 2500 μM (cyan) and 3000 μM (dark green) ncNTP. **H.** Model fit to H1242A misincorporation of 250 μM (red), 500 μM (blue), 750 μM (black), 1000 μM (light green), 1500 μM (magenta), 2000 μM (cyan) and 2500 μM (purple) ncNTP. **J.** The kinetic model predicted unusually high pyrophosphorolysis rate by WT RNAP of 1.9 s^{-1}. To test this prediction we analysed pyrophosphorolysis from the complex identical to the product complex of the model (panel A). We assumed that since PPi is released from the active centre slowly [1] the local concentration of PPi immediately after phosphodiester bond formation is maximal. We measured pyrophosphorolysis at varying PPi concentrations. Shown in the figure is the fit to the Michaelis-Menten equation. The k_{pol}(PPi) determined from the fit is 0.33 s^{-1} and K_M is 514 μM. Note however that the actual k_{ppi} is expected to be much higher than k_{pol}(PPi) since the reverse incorporation of NTP could not be excluded in this experiment (compare for example k_{pol} in Supplementary Table S1 to k_{pol} in Supplementary Table S3 for WT incorporation). Given this data, the WT pyrophosphorolysis rate predicted by the model seems reasonable.

SUPPLEMENTARY TABLE

Supplementary Table S1 <u>K_d and k_{pol} for incorporation and misincorporation by WT, ΔTL RNAP, N737A and ΔTL/N737A RNAPs</u>: Incorporation of cGTP was studied in EC^{G1}, misincorporation of ncGTP – in EC^{A}. K_d and k_{pol} (reaction rate at saturating NTP concentration) for cGTP incorporation by WT and N737A RNAPs were obtained by fitting the kinetics data into a simple kinetic model as described in Supplementary Methods. The rest of the data were fitted into the Michaelis-Menten equation as described in Materials and Methods.

RNAP	WT		N737A	
	k_{pol} (s^{-1})	K_d (μM)	k_{pol} (s^{-1})	K_d (μM)
cGTP	100	20	21	20
c2'dATP	$5.5 \pm 0.2 \times 10^{-2}$	400 ± 30	$1.8 \pm 0.1 \times 10^{-2}$	350 ± 40
c3'dATP	$1.4 \pm 0.1 \times 10^{-1}$	55 ± 8	$1.9 \pm 0.1 \times 10^{-1}$	43 ± 10
RNAP	ΔTL		ΔTL/N737A	
	k_{pol} (s^{-1})	K_d (μM)	k_{pol} (s^{-1})	K_d (μM)
cGTP	$1.6 \pm 0.1 \times 10^{-3}$	36 ± 6	$3.0 \pm 0.1 \times 10^{-3}$	96 ± 15
c2'dATP	$1.3 \pm 0.1 \times 10^{-3}$	50 ± 9	$1.1 \pm 0.1 \times 10^{-3}$	27 ± 6
c3'dATP	$2.7 \pm 0.2 \times 10^{-3}$	57 ± 11	$2.9 \pm 0.4 \times 10^{-3}$	60 ± 16

Supplementary Table S2 K_M^{obs} and k_{pol} for incorporation: Incorporation of cGTP was studied in EC^{G1}. K_M^{obs} and k_{pol} (reaction rate at saturating NTP concentration) for incorporation data of WT and mutants were obtained by a fit to the Michaelis-Menten equation as described in Experimental Procedures. The values for incorporation data for WT, R1239A and H1242A RNAPs are approximate since incorporation did not follow single exponential kinetics (Supplementary Methods for details).

	NTP incorporation	
RNAP	k_{pol} (s^{-1})	K_M^{obs} (μM)
WT	25.7 ± 2.4	19 ± 7
ΔTL	$1.6 \pm 0.1 \times 10^{-3}$	36 ± 6
WT/Stl	$3.6 \pm 0.2 \times 10^{-3}$	24 ± 6
H1242A	1.1 ± 0.1	91 ± 24
R1239A	1.9 ± 0.3	33 ± 21
H1242A/R1239A	$6.9 \pm 0.5 \times 10^{-2}$	37 ± 11
M1238A	$5.6 \pm 0.5 \times 10^{-2}$	47 ± 17

Supplementary Table S3 Rate parameters for the kinetic model. Table shows the kinetic rates for each step of the model (Supplementary Fig. S6A) for WT and mutant incorporation and misincorporation.

	k_b^+ (s^{-1})	k_b^- (s^{-1})	k^+ (μM^{-1} s^{-1})	k^- (s^{-1})	K_d (μM)	k_{pol} (s^{-1})	k_{ppi} (s^{-1})
WT	8.90	35.20	250	5000	20	100	1.98
H1242A	8.90	35.20	250	5000	20	9.77×10^{-1}	1×10^{-1}
R1239A	8.90	35.20	250	5000	20	2.1	2.99×10^{-1}
ΔTL	8.90	35.20	250	2500	10	1.9×10^{-3}	0
WT mis	8.90	35.20	250	625000	2500	9.11×10^{-2}	0
H1242A mis	8.90	35.20	250	625000	2500	6.4×10^{-3}	0
R1239A mis	8.90	35.20	250	625000	2500	9.0×10^{-3}	0

SUPPLEMENTARY REFERENCES

1. Johnson RS, Strausbauch M, Cooper R, Register JK: **Rapid kinetic analysis of transcription elongation by Escherichia coli RNA polymerase.** *J Mol Biol* 2008, **381:**1106-1113.
2. Gutenkunst RN, Casey FP, Waterfall JJ, Myers CR, Sethna JP: **Extracting falsifiable predictions from sloppy models.** *Ann N Y Acad Sci* 2007, **1115:**203-211.
3. Gutenkunst RN, Waterfall JJ, Casey FP, Brown KS, Myers CR, Sethna JP: **Universally sloppy parameter sensitivities in systems biology models.** *PLoS Comput Biol* 2007, **3:**1871-1878.
4. Temiakov D, Zenkin N, Vassylyeva MN, Perederina A, Tahirov TH, Kashkina E, Savkina M, Zorov S, Nikiforov V, Igarashi N, et al: **Structural basis of transcription inhibition by antibiotic streptolydigin.** *Mol Cell* 2005, **19:**655-666.

SUPPLEMENTARY FIGURES

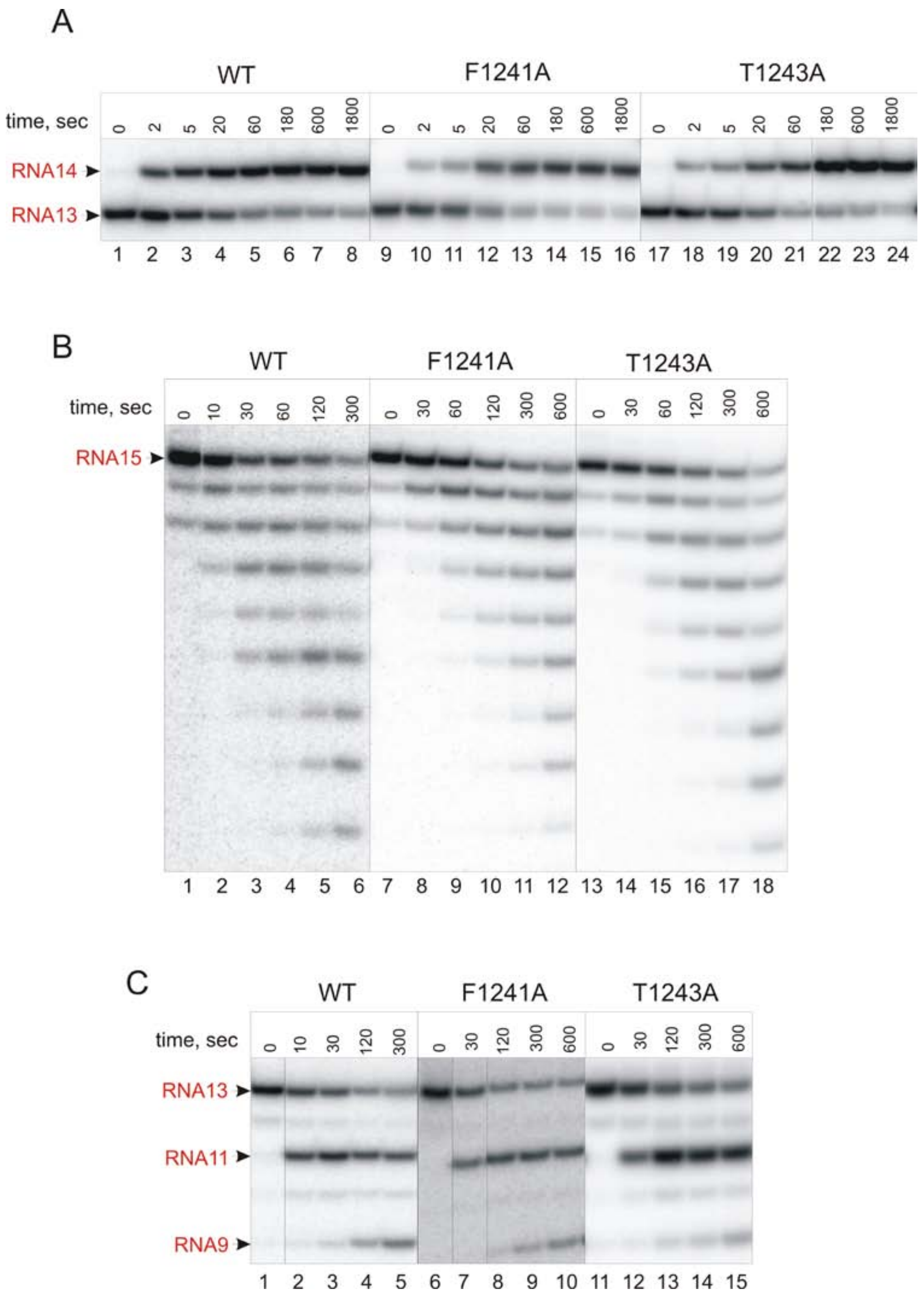

Supplementary Figure S1

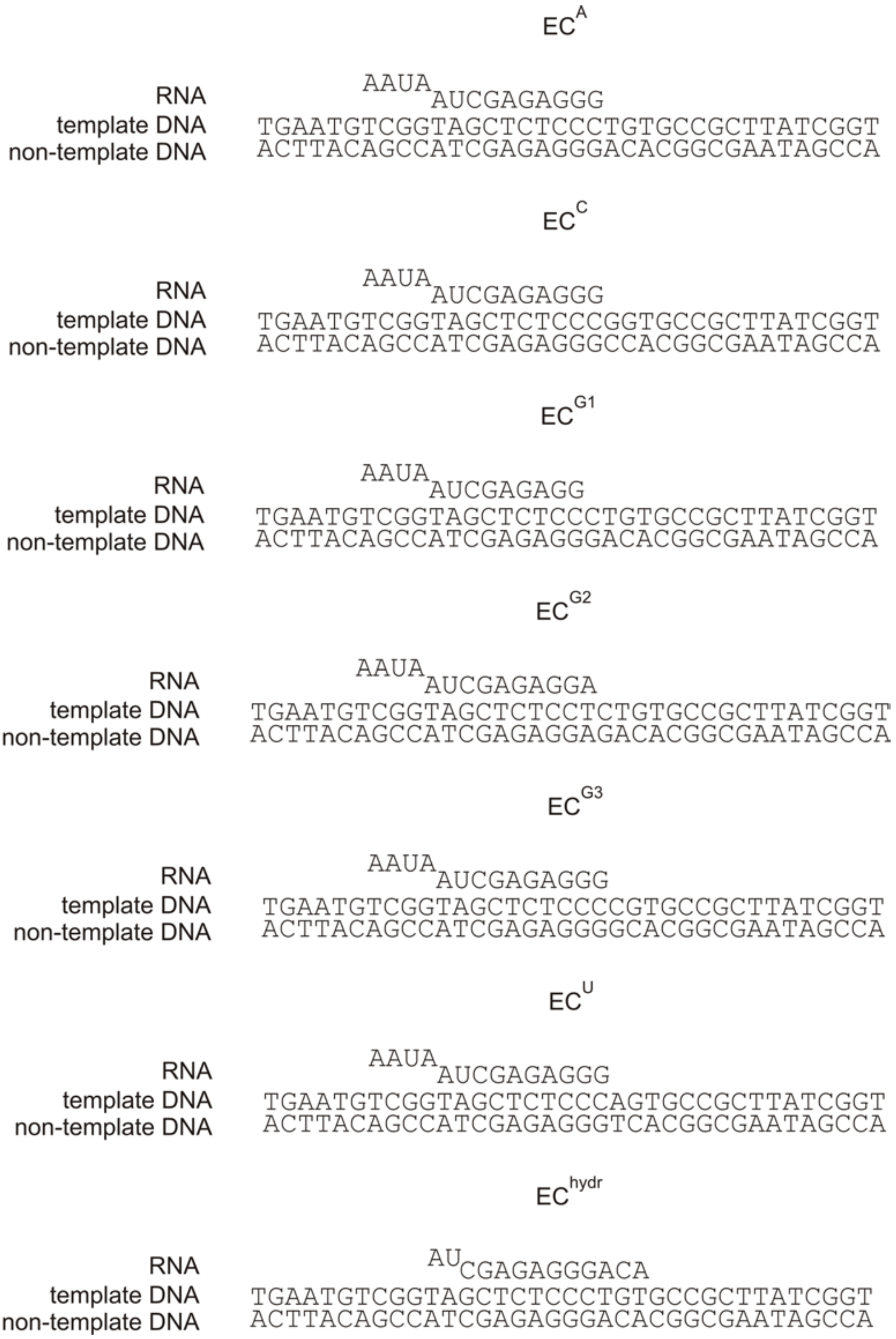

Supplementary Figure S2

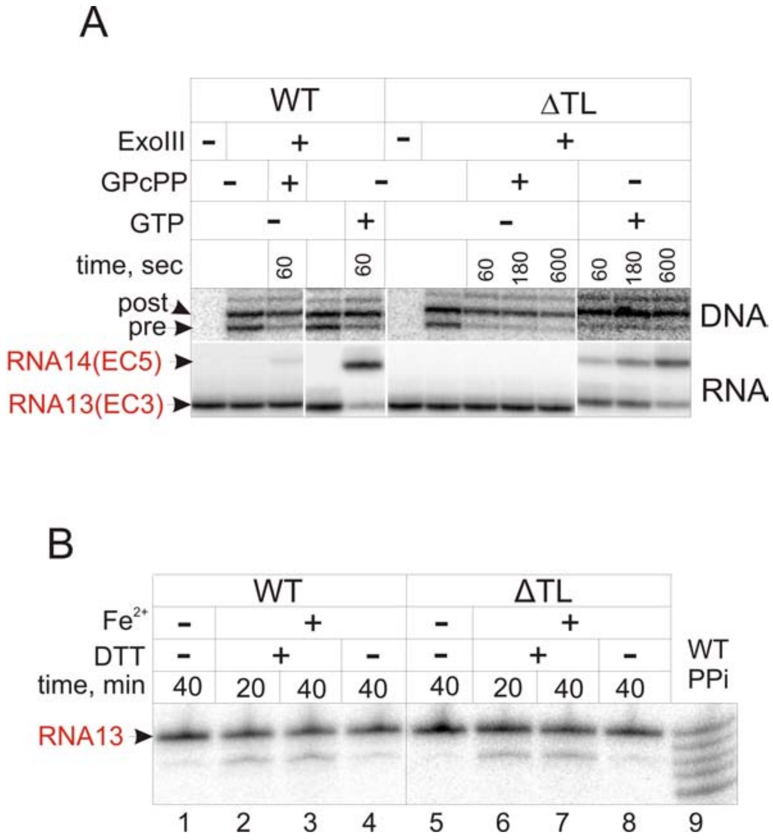

Supplementary Figure S3

EC^{G1}

ΔTL RNAP k_{obs}[1mM cGTP]

RNA	AAUA AUCGAGAGG	
template DNA	TGAATGTCGGTAGCTCTCCCTGTGCCGCTTATCGGT	$1.6 \pm 0.1 \times 10^{-3}\ s^{-1}$
non-template DNA	ACTTACAGCCATCGAGAGGGACACGGCGAATAGCCA	

EC^{G3}

RNA	AAUA AUCGAGAGGG	
template DNA	TGAATGTCGGTAGCTCTCCCCGTGCCGCTTATCGGT	$2.7 \pm 0.2 \times 10^{-3}\ s^{-1}$
non-template DNA	ACTTACAGCCATCGAGAGGGGCACGGCGAATAGCCA	

EC^{A}

ΔTL RNAP k_{obs}[1mM cATP]

RNA	AAUA AUCGAGAGGG	
template DNA	TGAATGTCGGTAGCTCTCCCTGTGCCGCTTATCGGT	$8.1 \pm 0.5 \times 10^{-3}\ s^{-1}$
non-template DNA	ACTTACAGCCATCGAGAGGGACACGGCGAATAGCCA	

Supplementary Figure S4

A *E.coli* ΔTL, 1 mM ncGTP

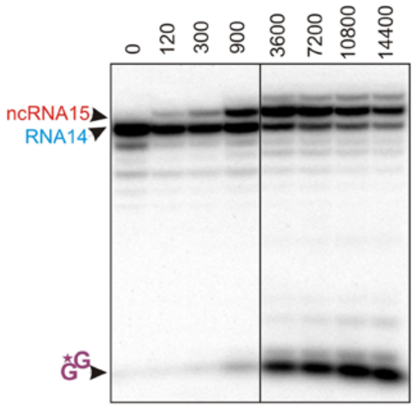

B

E.coli ΔTL RNAP (rate in saturating substrates)		discrimination against	(discrimination against by *T.aquaticus* ΔTL RNAP)
cGTP (EC^{G3})	0.023 s^{-1}		
ncGTP (EC^{A})	0.001 s^{-1}	~25	~6
cATP (EC^{A})	0.04 s^{-1}		
c2'dATP (EC^{A})	0.0027 s^{-1}	~15	~6
c3'dATP (EC^{A})	0.0025 s^{-1}	~15	~3

Supplementary Figure S5

A

$$Pre \underset{k_b^-}{\overset{k_b^+}{\rightleftharpoons}} Post \underset{k^-}{\overset{k^+}{\rightleftharpoons}} Post \cdot [NTP] \underset{k_{ppi}}{\overset{k_{cat}}{\rightleftharpoons}} P \cdot PPi$$

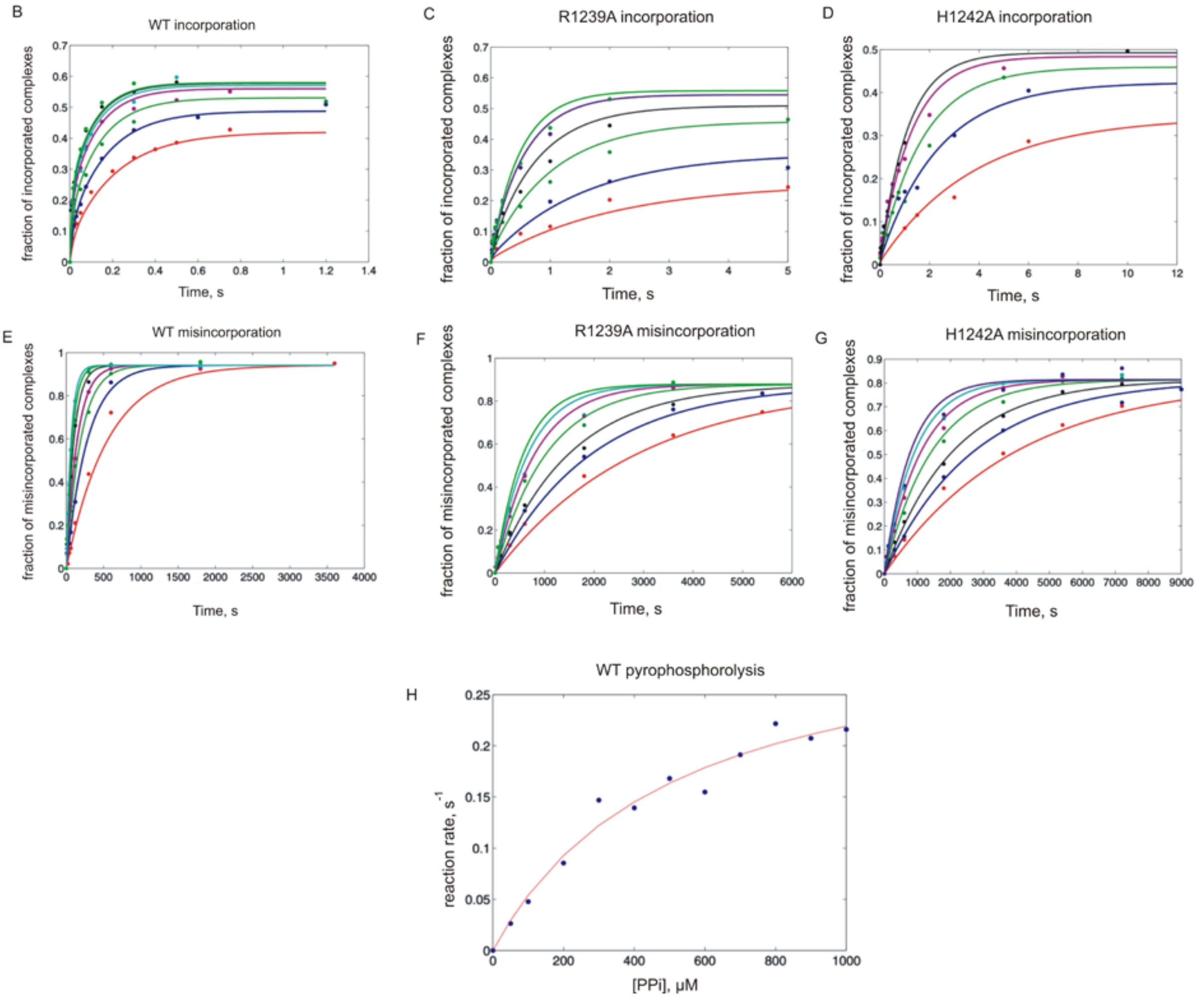

Supplementary Figure S6
